# Indoxyl Sulfate Downregulates Expression of Mas Receptor via OAT3/AhR/Stat3 Pathway in Proximal Tubular Cells

**DOI:** 10.1371/journal.pone.0091517

**Published:** 2014-03-10

**Authors:** Hwee-Yeong Ng, Maimaiti Yisireyili, Shinichi Saito, Chien-Te Lee, Yelixiati Adelibieke, Fuyuhiko Nishijima, Toshimitsu Niwa

**Affiliations:** 1 Department of Advanced Medicine for Uremia, Nagoya University Graduate School of Medicine, Nagoya, Japan; 2 Division of Nephrology, Department of Internal Medicine, Kaohsiung Chang Gung Memorial Hospital and Chang Gung University College of Medicine, Kaohsiung, Taiwan; 3 Biomedical Research Laboratories, Kureha Co., Tokyo, Japan; National Cancer Institute, United States of America

## Abstract

Renin-angiotensin system (RAS) plays a pivotal role in chronic kidney disease (CKD). Angiotensin converting enzyme-related carboxypeptidase 2 (ACE2)/angiotensin (Ang)-(1–7)/Mas receptor axis counteracts the deleterious actions of Ang II. ACE2 exerts its actions by cleaving Ang II into Ang-(1–7) which activates Mas receptor. This study aimed to determine if the expression of Mas receptor is altered in the kidneys of CKD rats, and if indoxyl sulfate (IS), a uremic toxin, affects the expression of Mas receptor in rat kidneys and cultured human proximal tubular cells (HK-2 cells). The expression of Mas receptor was examined in the kidneys of CKD and AST-120-treated CKD rats using immunohistochemistry. Further, the effects of IS on Mas receptor expression in the kidneys of normotensive and hypertensive rats were examined. The effects of IS on the expression of Mas receptor and phosphorylation of endothelial nitric oxide synthase (eNOS) in HK-2 cells were examined using immunoblotting. CKD rats showed reduced renal expression of Mas receptor, while AST-120 restored its expression. Administration of IS downregulated Mas receptor expression in the kidneys of normotensive and hypertensive rats. IS downregulated Mas receptor expression in HK-2 cells in a time- and dose-dependent manner. Knockdown of organic anion transporter 3 (OAT3), aryl hydrocarbon receptor (AhR), and signal transducer and activator of transcription 3 (Stat3) inhibited IS-induced downregulation of Mas receptor and phosphorylated eNOS. N-acetylcysteine, an antioxidant, also inhibited IS-induced downregulation of Mas receptor and phosphorylated eNOS. Ang-(1–7) attenuated IS-induced transforming growth factor-β1 (TGF-β1) expression.

**Conclusion:**

Mas receptor expression is reduced in the kidneys of CKD rats. IS downregulates renal expression of Mas receptor via OAT3/AhR/Stat3 pathway in proximal tubular cells. IS-induced downregulation of Mas receptor might be involved in upregulation of TGF-β1 in proximal tubular cells.

## Introduction

Renin-angiotensin system (RAS) plays a pivotal role in chronic kidney disease (CKD). RAS is regulated and modulated by two axes. The first axis primarily consists of angiotensin converting enzyme (ACE), angiotensin (Ang) II and Ang II type 1 receptor. This axis induces vasoconstriction, proliferation and oxidative stress [1]. The second axis, which consists of angiotensin converting enzyme-related carboxypeptidase 2 (ACE2), Ang-(1–7), and Mas receptor, counteracts the deleterious actions of Ang II as an endogenous regulator of RAS. ACE2 degrades Ang II into Ang-(1–7), which binds to Mas receptor [2], and then induces vasodilatation, and attenuates inflammation and fibrosis [3]. Mas receptor deficiency mice demonstrated a variety of complications including metabolic syndrome-like state, impaired heart function, increased blood pressure, and endothelial dysfunction [4]. The ACE2/Ang-(1–7)/Mas axis is protective in renal disease [5], [6], proven by the transgenic and knockout mouse models. However, the expression of ACE2 in diseased kidneys in previous studies is divergent [7]. In addition, there are controversial data on the renoprotective effect of Ang-(1–7), including stimulation of inflammation by Ang-(1–7) [8]. Little is known about Mas receptor expression in CKD. Indoxyl sulfate (IS), one of protein-bound uremic toxins, elicits a variety of cytotoxic effects, and exacerbates CKD [9], [10]. The role of IS on the Mas receptor has not been elucidated. This study aimed to determine if the expression of Mas receptor is altered in the kidneys of CKD rats, and if IS affects the expression of Mas receptor in rat kidneys and cultured human proximal tubular cells.

## Materials and Methods

### Reagents

Antibodies were obtained from the following suppliers: anti-Mas receptor (AAR-013) (Alomone Laboratories, Ltd., Jerusalem, Israel); anti-endothelial nitric oxide synthase (eNOS) (BD Biosciences, Mississauga, ON, Canada); anti-aryl hydrocarbon receptor (AhR) (Santa Cruz Biotechnology, Santa Cruz, CA, USA); anti-α-tubulin (Calbiochem, La Jolla, CA, USA); anti-signal transducer and activator of transcription 3 (Stat3), anti-phosphorylated eNOS (Ser1177) (peNOS), anti-transforming growth factor-β1 (TGF-β1), anti-rabbit IgG horseradish peroxidase-linked antibody and anti-mouse IgG horseradish peroxidase-linked antibody (Cell Signaling Technology, Beverly, MA, USA). IS was obtained from Alfa Aesar (Morecambe, Lancs., UK). Ang-(1–7) was purchased from Bachem California (Torrance, CA, USA). *N*-acetylcysteine (NAC), an antioxidant, was from Calbiochem (La Jolla, CA, USA). Dulbecco’s modified Eagle’s medium/F12 was purchased from Wako (Osaka, Japan). Trypsin-EDTA, fetal bovine serum, and insulin transferrin-selenium were purchased from Gibco (Grand Island, NY, USA). Penicillin and streptomycin were purchased from Nacalai Tesque Inc. (Kyoto, Japan).

### Cell Culture

HK-2 cells derived from human proximal tubular cells were purchased from ATCC (Manassas, VA, USA). The cells were incubated at 37°C under 5% CO_2_ humidified atmosphere, and were maintained in Dulbecco’s modified Eagle’s medium/F12 supplemented with 10% FBS, insulin-transferrin-selenium, 100 U/mL penicillin, and 100 µg/mL streptomycin.

HK-2 cells were starved in Dulbecco’s modified Eagle’s medium/F12 for 24 h before stimulation. In the time course-experiment, HK-2 cells were incubated with IS (250 µM) for 1, 3, 6, 12, 24, or 48 h. In the dose-experiment, the cells were incubated with IS at a concentration of 0, 50, 100, 200, or 250 µM. In the experiments with NAC and Ang-(1–7), the cells were incubated with or without NAC (5 mM) or Ang-(1–7) (10^−8^∼10^−6 ^M) for 30 min followed by indoxyl sulfate (250 µM) for 48 h (for Mas receptor) or 72 h (for TGF-β1).

### Preparation of Small Interfering RNAs Specific to OAT3, AhR, and Stat3

Small interfering RNAs (siRNAs) specific to Stat3 were obtained from Nippon Gene Material (Tokyo, Japan). The sense sequences of the Stat3 siRNAs were 5-GGAGCAGCACCUUCAGGAUdTdT-3. OAT3 and AhR siRNA were purchased from Santa Cruz Biotechnology (Santa Cruz, CA, USA). Lipofectamin RNAiMAX (Invitrogen, Life Technologies, Carlsbad, CA, USA) was used to transfect siRNAs into HK-2 cells according to the manufacturer’s protocol. HK-2 cells were treated with or without OAT3 (10 nM), AhR siRNA (30 nM) or Stat3 siRNA (10 nM) for 24 h. Then, the cells were washed twice with PBS, and lysed in the lysis buffer.

### Animal Experiments

The following animal studies were approved by Animal Care Committee of Biomedical Research Laboratories of Kureha, and were performed according to the Guiding Principles for the Care and Use of Laboratory Animals of the Japanese Pharmacological Society.

#### Animal study 1

Experimental rats were produced as reported previously [11]. Briefly, 7-week-old male Sprague-Dawley rats (Clea, Tokyo, Japan) were used to produce CKD by 5/6-nephrectomy. In the first session, while the renal artery and vein of the left kidney were ligated, two-thirds of the left kidney was removed with a razor, and thrombin was applied onto the cut surface for hemostasis. Then, the artery and vein of the left kidney were unligated. One week after the first operation, the right kidney was removed after ligation of the renal artery and vein. These operations were performed under anesthesia with sodium pentobarbital (Schering-Plough, Corp., NJ, USA). Eleven weeks after 5/6-nephrectomy, the rats were randomized into two groups CKD (n = 8), and AST-120-treated CKD rats (n = 8). AST-120 was administered to the rats at a dose of 4 g/kg with powder chow (CE-2, Clea) for 16 weeks, whereas powder chow alone was administered to control and CKD rats. Normal rats (n = 9) were used as a control group.

#### Animal study 2

To test the direct effect of IS on Mas receptor, animals with normal renal function were fed with or without IS (200 mg/kg of IS in drinking water). Experimental rats were produced as reported previously [12]. Briefly, 5-week-old male Dahl salt-sensitive rats (Dahl-Iwai S, n = 32) were purchased from Japan SLC (Hamamatsu, Shizuoka, Japan), and were fed with powder rat chow (CE-2; Clea, Tokyo, Japan) and water. At 16th week of age, the rats were divided into four groups: 1) Dahl normotensive rats (DN), and 2) Dahl normotensive indoxyl sulfate-administered rats (DN+IS; 200 mg/kg/day of indoxyl sulfate in drinking water), 3) Dahl hypertensive rats (DH), and 4) Dahl hypertensive indoxyl sulfate-administered rats (DH+IS). After 32 weeks, blood pressure was measured using the tails of the rats with a pneumatic cuff and a sphygmomanometer for small animals (UR-5000, Ueda Avancer Co., Tokyo, Japan), then the rats were anesthetized, and renal cortices were isolated.

### Immunohistochemistry

Immunohistochemistry was performed using the streptavidin-biotin complex method. Paraffin-embedded fixed tissue sections (4 µm) were deparaffinized with xylene, and rehydrated with ethanol. Antigen retrieval was carried out with 10 mM citrate buffer (pH 6.0) twice for 5 min microwave treatment. The sections were incubated with 3% H_2_O_2_ methanol for 10 min, and then incubated with 10% serum (Nichirei Co., Tokyo, Japan) for 30 min at room temperature. After that, anti-Mas receptor antibody (1∶100) was added, and incubated at 4°C overnight. The next day, the sections were incubated with the secondary antibody at room temperature for 30 min, and then with peroxidase-conjugated streptavidin (Nichirei Co.) at 37°C for 30 min. The localization of Mas receptor was visualized using 3,3′-diaminobenzidine tetrahydrochloride (Merck KGaA, Darmstadt, Germany) at a concentration of 30 mg/mL, containing 0.03% H_2_O_2_. The sections were photographed (DN100, E600; Nikon, Tokyo, Japan). The immunostaining-positive areas were then quantified in 20 random renal cortex sections using NIH Image 1.62.

### Immunoblotting

Immunoblotting was performed as described previously [13], [14]. In brief, cell lysates were fractionated by sodium dodecyl sulfate-polyaclylamide gel electrophoresis (SDS-PAGE) on polyacrylamide gels (8∼12%), and proteins were transferred to polyvinylidene fluoride membranes (Immobilon-P, Millipore, Bedford, MA, USA). Mas receptor, peNOS, AhR, OAT3, Stat3, and TGF-β1 were detected using their specific antibodies. To normalize the blots for protein levels, after being immunoblotted with the specific antibodies, the blots were stripped and reprobed with either anti-α-tubulin or anti-eNOS antibodies (for peNOS). The protein bands were visualized using the enhanced Chemi-Lumi One system (Nacalai Tesque).

### Statistical Analysis

Data analysis was performed with SPSS Statistics version 17 (IBM, Armonk, NY, USA). Results are expressed as mean±SE. Student t-test was used to analyze the difference of mean values between two groups. Comparison among different groups was performed by using one-way analysis of variance (ANOVA), and then examined by least significance difference (LSD) test. A *p* value <0.05 is considered to be statistically significant.

## Results

### Mas Receptor Expression is Reduced in Kidneys of CKD Rats

**Figure 1:**
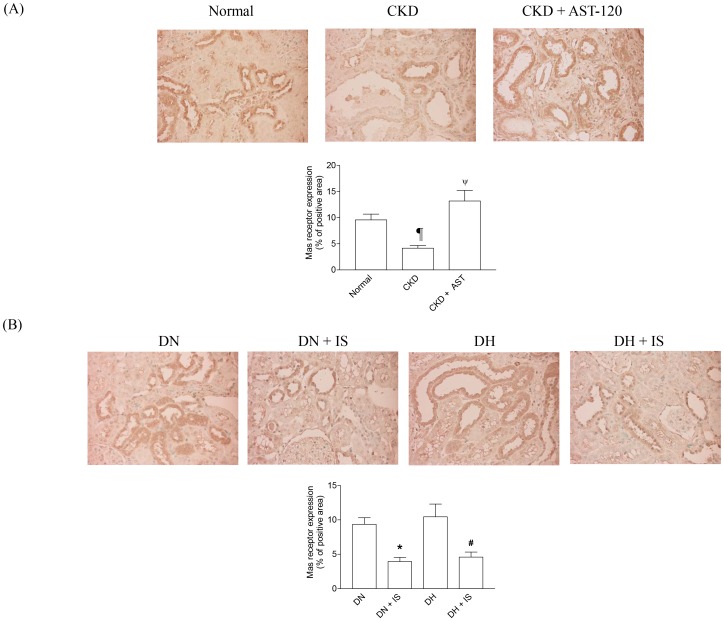
Immunohistochemical staining of Mas receptor in kidneys of CKD and Dahl rats. CKD rats showed significantly lower level of Mas receptor expression than normal. By administrating AST-120, Mas receptor expression was restored (A). The expression of Mas receptor was downregulated by IS in normotensive and hypertensive Dahl rats with normal renal function (B). The pictures were taken under ×400 magnification (n = 8 for each group). Data are expressed as mean±SE. ¶p<0.05 *vs.* normal; ψp<0.05 *vs.* CKD; *p<0.05 *vs.* DN; #p<0.05 *vs.* DH.

We first examined whether the expression of Mas receptor is altered in the kidneys of CKD rats, and whether AST-120, an oral sorbent, affects its expression. AST-120 reduces the serum level of indoxyl sulfate by adsorbing its precursor, indole, in the intestine [10], [15], [16]. Laboratory parameters of these rats, which have been reported previously [11], are cited in Table 1. Immunohistochemisty revealed that Mas receptor was mainly localized in renal proximal tubular cells (Fig. 1). CKD rats showed significantly decreased Mas receptor-positive area in the kidney as compared with normal (Fig. 1A). However, AST-120 treatment significantly alleviated the decrease of Mas receptor-positive area in the kidney (Fig. 1A). Thus, the expression of Mas receptor was decreased in the kidneys of CKD rats, and AST-120 restored its expression.

**Table 1:** Biochemical data of the animals at the end of the study.

Animal study 1	Normal	CKD	CKD+AST-120	
	(n = 9)	(n = 8)	(n = 8)	
Serum creatinine (mg/dL)	0.40±0.01	1.41±0.23¶	1.17±0.06	
Creatinine clearance (mL/min)	3.68±0.13	1.14±0.16¶	1.20±0.09	
Serum indoxyl sulfate (mg/dL)	0.08±0.007	0.52±0.16¶	0.12±0.02^ψ^	
**Animal study 2**	**DN**	**DN+IS**	**DH**	**DH+IS**
	**(n = 8)**	**(n = 8)**	**(n = 8)**	**(n = 8)**
Systolic blood pressure (mmHg)	143±3	141±3	158±5	158±9
Serum creatinine (mg/dL)	0.56±0.01	0.58±0.01	0.55±0.01	0.58±0.01
Creatinine clearance (mL/min)	1.91±0.09	1.81±0.07	1.90±0.09	1.79±0.05
Serum indoxyl sulfate (mg/dL)	0.10±0.01	0.94±0.13*	0.06±0.01	1.89±0.26^#^

Data were cited from reference [11] for animal study 1 and [12] for animal study 2.

Data are expressed as mean±SE.

Abbreviation: CKD, chronic kidney disease; DN, Dahl normotensive rats; DH, Dahl hypertensive rats; IS, indoxyl sulfate.

¶ p<0.05 *vs*. normal, ^ψ^p<0.05 *vs*. CKD.

*p<0.05 *vs.* DN, ^#^p<0.05 *vs.* DH.

### Administration of Indoxyl Sulfate Reduces Mas Receptor Expression in Rat Kidneys

We next determined whether administration of indoxyl sulfate reduces the expression of Mas receptor in rat kidneys. Laboratory parameters as reported previously [12] are cited in Table 1. Both DN+IS and DH+IS rats showed significantly decreased Mas receptor-positive areas as compared with DN and DH rats, respectively (Fig. 1B). However, there was no significant difference in Mas receptor-positive area between DN and DH groups (Fig. 1B). Thus, administration of indoxyl sulfate reduced Mas receptor expression in the kidneys of normotensive and hypertensive rats.

### Indoxyl Sulfate Downregulates Mas Receptor Expression in Proximal Tubular Cells

**Figure 2:**
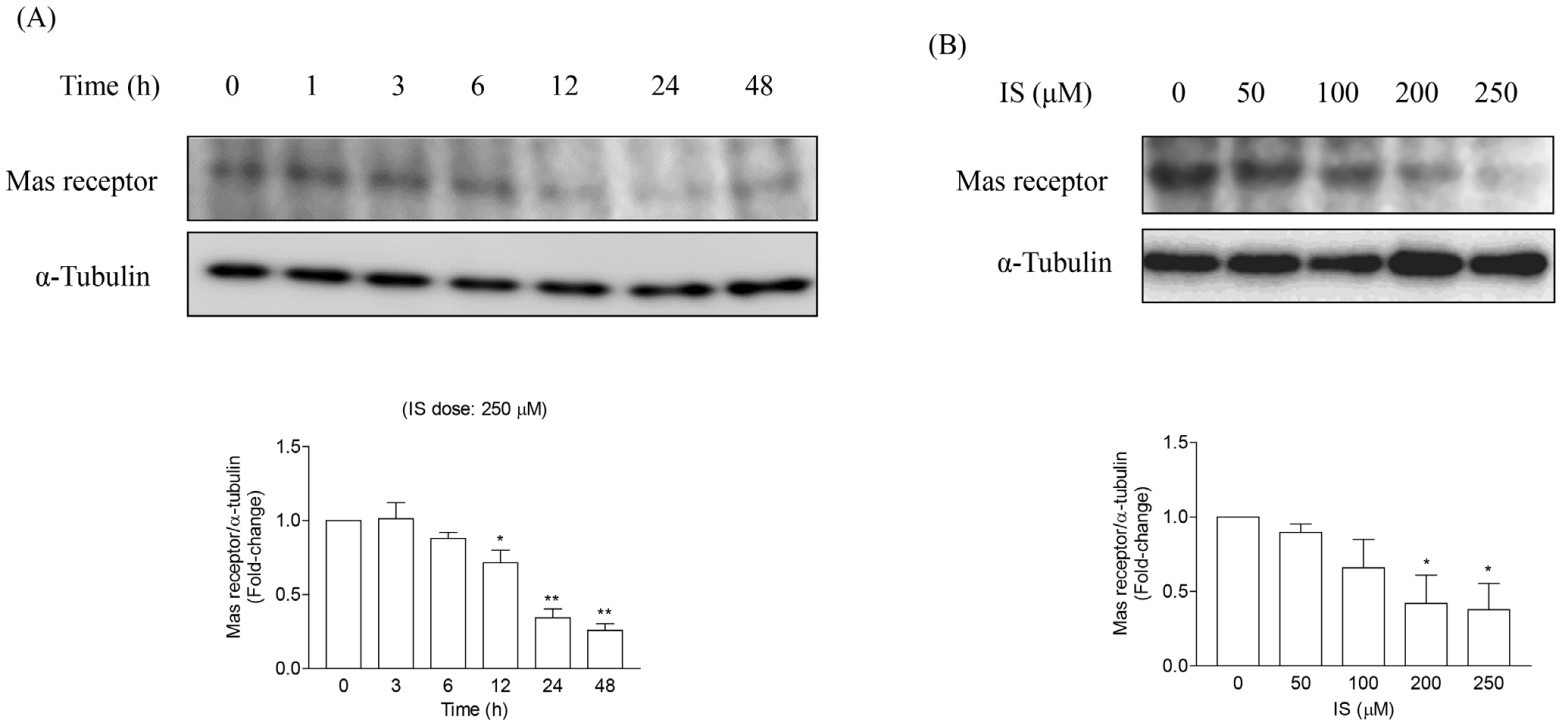
Time- and dose-dependent effects of IS on Mas receptor protein expression in HK-2 cells. Mas receptor in the HK-2 was reduced by IS in a time- (A) and dose- (B) dependent manner. Bars represent means±SE, expressed as relative change in comparison with the basal value (n*≥3* for every experiment).*p<0.05 *vs.* basal value; **p<0.001 *vs.* basal value.

We examined the effects of IS on Mas receptor expression in HK-2 cells at different time durations and doses. IS reduced the expression of Mas receptor in a time- and dose-dependent manner (Fig. 2). IS at a concentration of 250 µM, which is comparable to its average serum level observed in uremic patients [10], decreased Mas receptor expression.

### Accumulation of IS via OAT3 in Proximal Tubular Cells Downregulates Mas Receptor Expression

**Figure 3:**
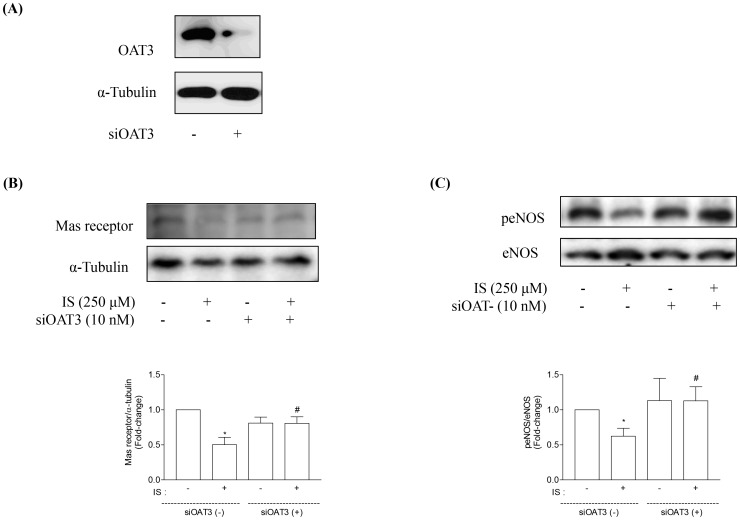
Effects of OAT3 siRNA on Mas receptor and peNOS expression in HK-2 cells. HK-2 cells were treated with or without OAT3 siRNA (siOAT3, 10 nM) (A). Mas receptor (B) and peNOS (C) protein expression was abolished by knocking down OAT3. Data are means±SE, expressed as relative change in comparison with the basal value (n*≥3* for every experiment). *p<0.05 *vs.* control; **#**p<0.05 *vs.* IS.

We investigated the mechanism how IS induced downregulation of Mas receptor. OAT3 is the major transporter for transcellular transport of IS into proximal tubular cells [17]. To confirm the effect of IS on Mas receptor, we also investigated the alteration of peNOS, because Mas receptor activates Akt/eNOS pathway [18] by phosphorylation of eNOS. Under physiological conditions, renal nitric oxide (NO) is derived mainly from constitutive NOS, including eNOS and neuronal NOS [19]. HK-2 cells were treated with or without 10 nM OAT3 siRNA for 24 h (Fig. 3A). IS reduced the expression of Mas receptor and peNOS (Fig. 3B, C). Knockdown of OAT3 blocked the inhibitory effects of IS on Mas receptor and peNOS (Fig. 3B, C).

### AhR and Stat3 are Involved in IS-induced Downregulation of Mas Receptor in Proximal Tubular Cells

**Figure 4:**
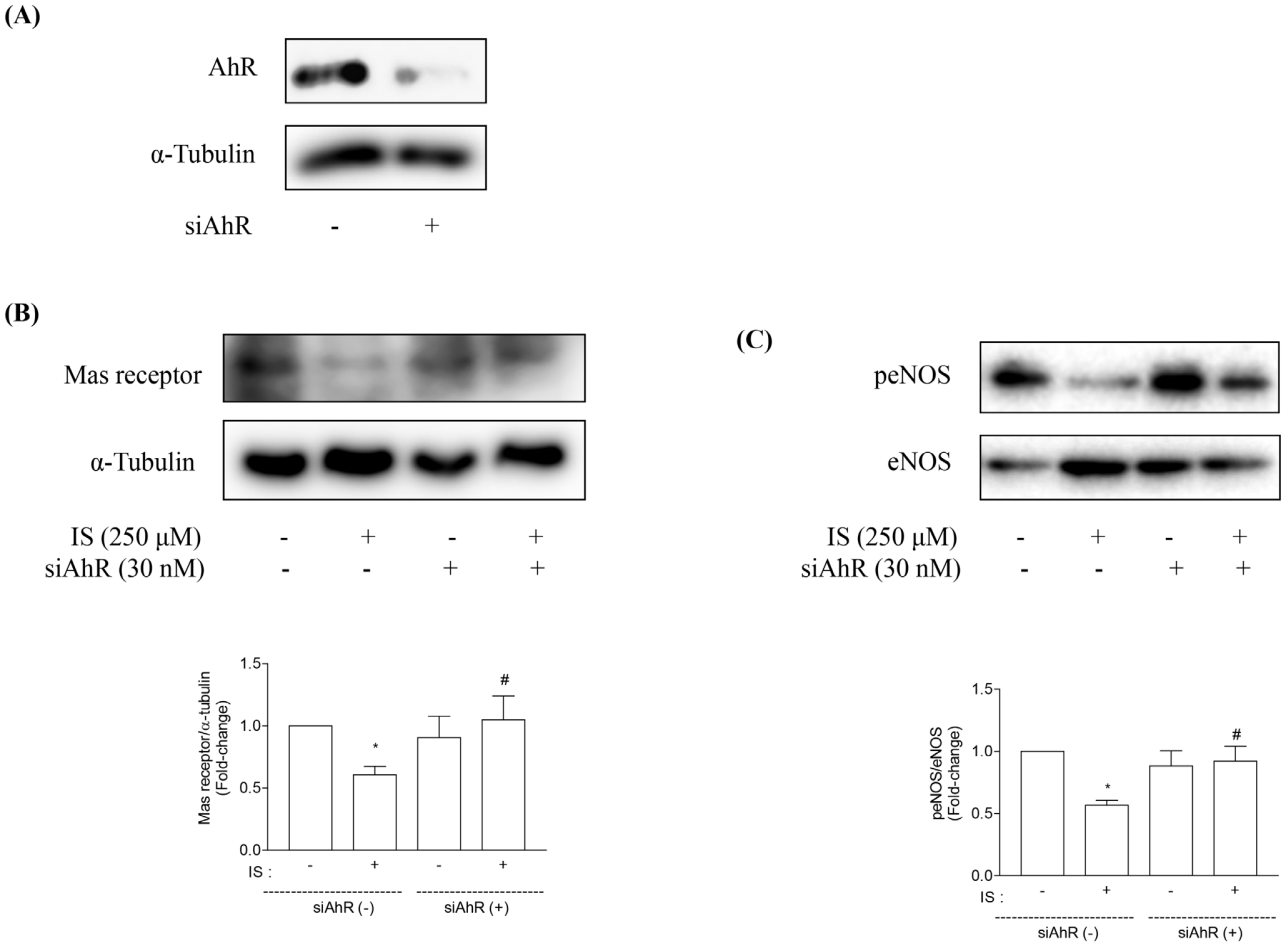
Effects of AhR siRNA on Mas receptor and peNOS expression in HK-2 cells. HK-2 cells were treated with or without AhR siRNA (siAhR, 30 nM) (A). Knockdown of AhR inhibited IS-induced downregulation of Mas receptor (B) and peNOS (C) expression. Data are means±SE, expressed as relative change in comparison with the basal value (n*≥3* for every experiment). *p<0.05 *vs.* control; **#**p<0.05 *vs.* IS.

AhR has been demonstrated to form a complex with IS in cytoplasm [20]. HK-2 cells were treated with or without 30 nM AhR siRNA for 24 h (Fig. 4A). Knockdown of AhR blocked the inhibitory effects of IS on Mas receptor and peNOS (Fig. 4B, C).

**Figure 5:**
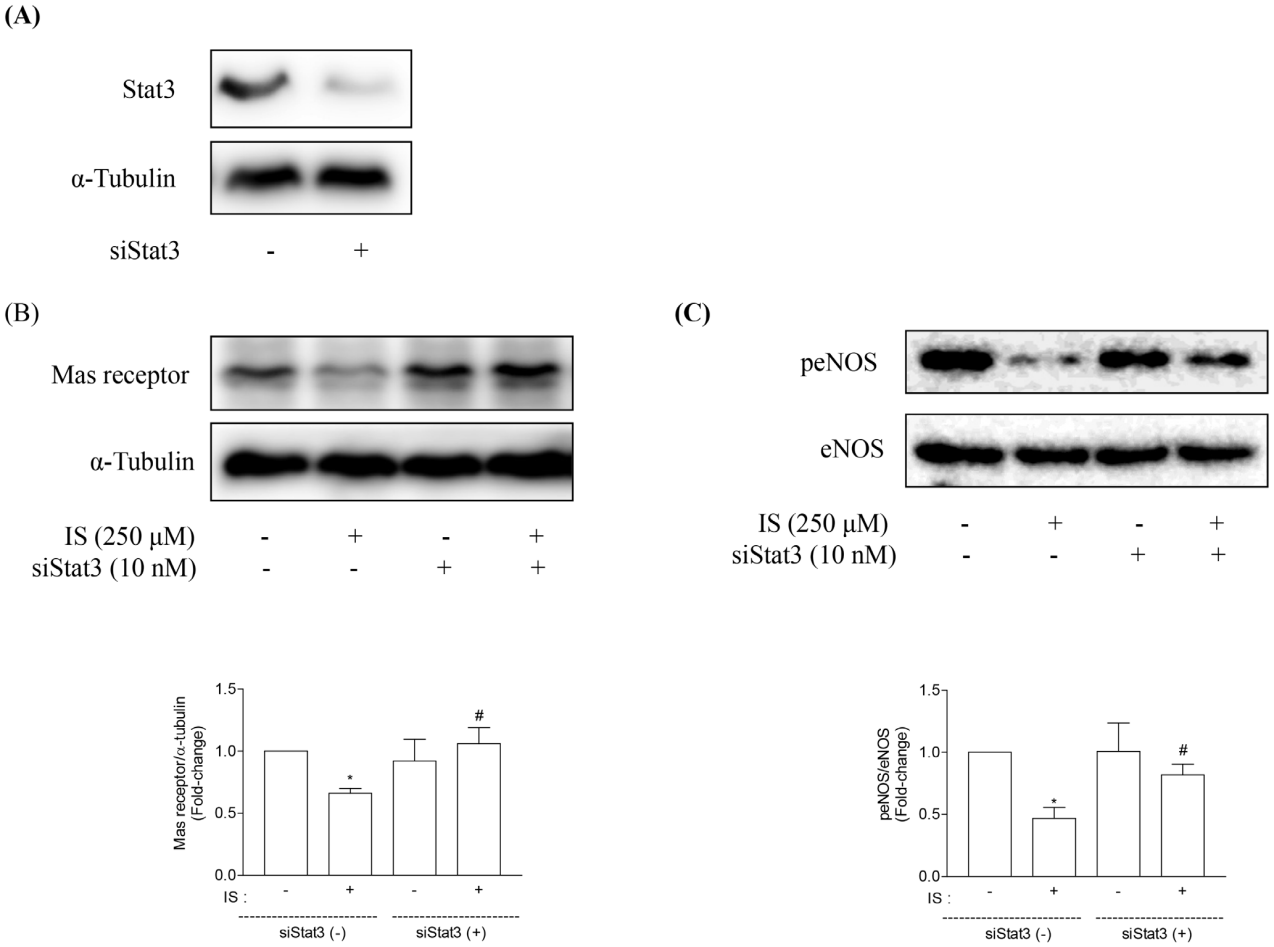
Effects of Stat3 siRNA on Mas receptor and peNOS expression in HK-2 cells. HK-2 cells were treated with or without Stat3 siRNA (siStat3, 10 nM) (A). Knockdown of Stat3 inhibited IS-induced downregulation of Mas receptor (B) and peNOS (C) expression. Data are means±SE, expressed as relative change in comparison with the basal value (n*≥3* for every experiment). *p<0.05 *vs.* control; #p<0.05 *vs.* IS.

Furthermore, AhR interacts with the other transcription factors such as Stat3 [21] in the cytoplasm after binding with its ligand. Previously, we have demonstrated that Stat3 is involved in IS-induced fibrogenesis and inflammation [22]. We then investigated the role of Stat3 in the downregulation of Mas receptor. HK-2 cells were treated with or without 10 nM Stat3 siRNA (Fig. 5A). Knockdown of Stat3 blocked the inhibitory effects of IS on Mas receptor and peNOS (Fig. 5B, C).

### Reactive Oxygen Species are Involved in IS-induced Downregulation of Mas Receptor in Proximal Tubular Cells

**Figure 6:**
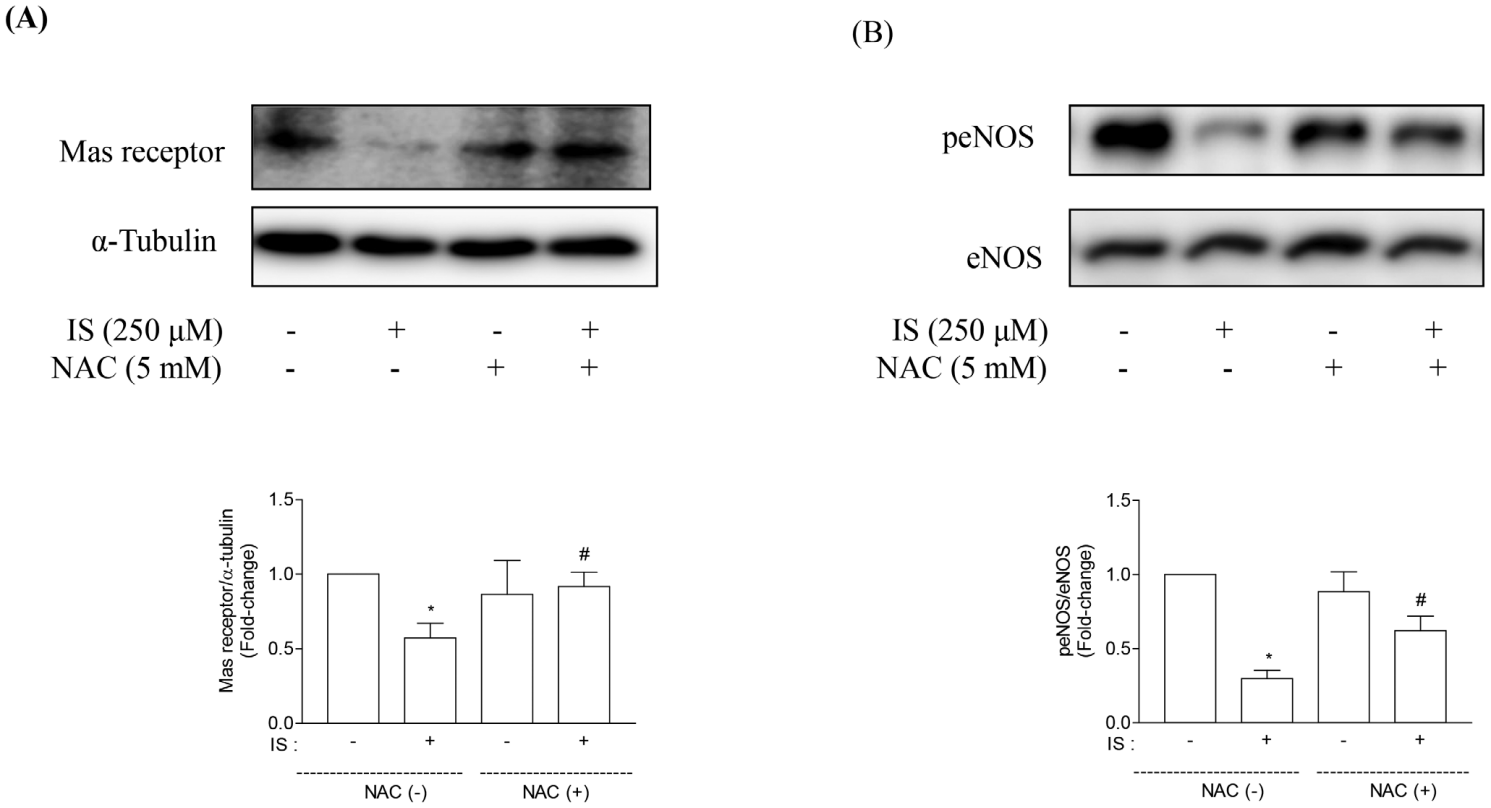
Effects of NAC on Mas receptor and peNOS expression in HK-2 cells. NAC (5 mM) inhibited IS-induced downregulation of Mas receptor (A) and peNOS (B) expression. Data are means±SE, expressed as relative change in comparison with the basal value (n*≥3* for every experiment). *p<0.05 *vs.* control; #p<0.05 *vs.* IS.

To study the influence of reactive oxygen species on IS-induced downregulation of Mas receptor, NAC, an antioxidant, was used. NAC blocked the inhibitory effects of IS on Mas receptor and peNOS (Fig. 6A, B). Thus, reactive oxygen species are also involved in IS-induced downregulation of Mas receptor.

### Activation of Mas Receptor by Ang-(1–7) Inhibits IS-induced Production of TGF-β1 in Proximal Tubular Cells

**Figure 7:**
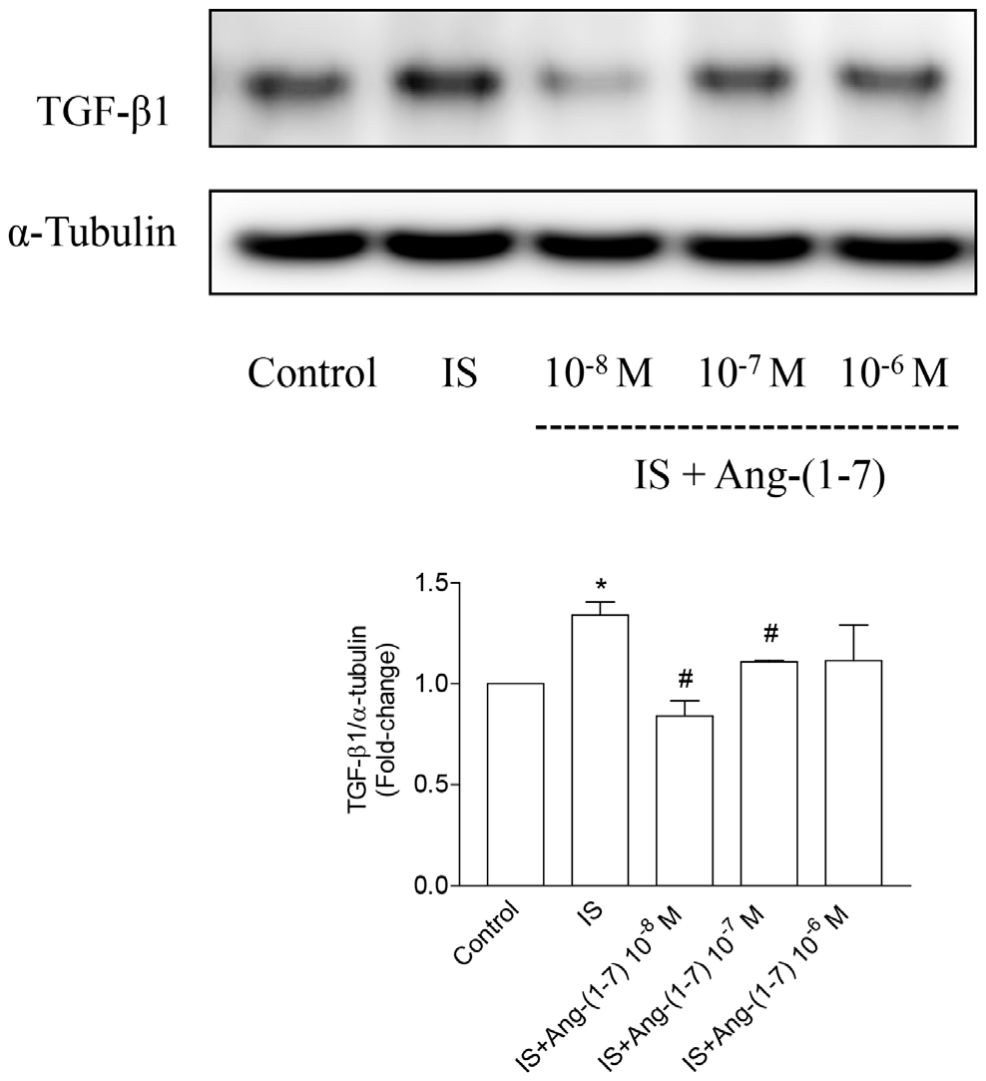
Effect of Ang-(1–7) on TGF-β1 expression in IS-treated HK2 cells. IS enhanced the expression of TGF-β1, whereas Ang-(1–7) inhibited it. Data are means±SE, expressed as relative change in comparison with the basal value (n*≥3* for every experiment). *p<0.05 *vs.* control; #p<0.05 *vs.* IS.

It is unclear whether activation of Mas receptor could provide beneficial effects on lowering renal toxicity of IS. We examined the effects of Ang-(1–7) on TGF-β1 expression in HK-2 cells treated with IS for 72 h. IS significantly upregulated expression of TGF-β1 (Fig. 7). Pretreatment of Ang-(1–7) at a concentration of 10^−8^ and 10^−7^ M significantly inhibited IS-induced upregulation of TGF-β1 (Fig. 7). Thus, activation of Mas receptor by Ang-(1–7) inhibits IS-induced upregulation of TGF-β1.

## Discussion

**Figure 8:**
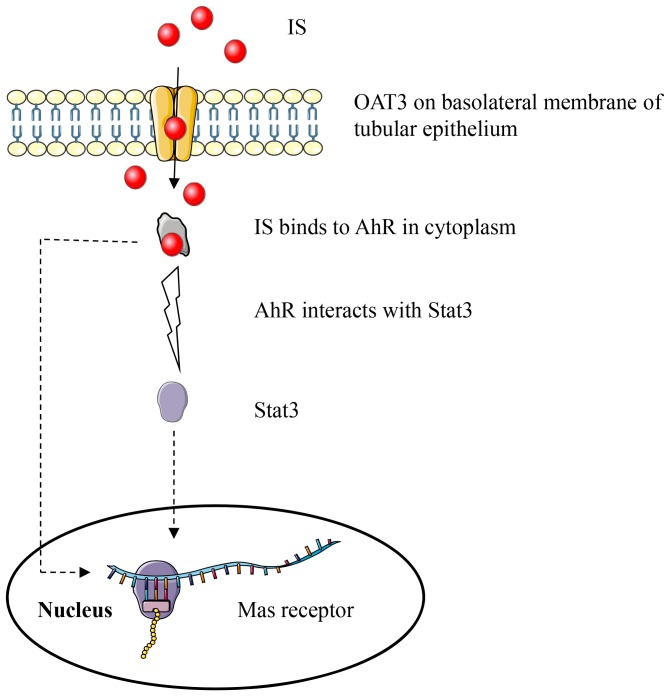
Schema of mechanism of IS-induced Mas receptor downregulation. IS accumulates in HK-2 cells via OAT3. In the cells, IS acts as a ligand of AhR. The IS-AhR complex then interacts with Stat3. In turn, Mas receptor is downregulated by IS-AhR-Stat3 or IS-AhR complex. This figure was created using Servier Medical Art (www.servier.com).

The main pathophysiological mechanism associated with CKD results from the activation of RAS. IS upregulates most of the ACE/Ang II/Ang II type 1 receptor axis components, including renin, angiotensinogen and Ang II type 1 receptor, but downregulates Ang II type 2 receptor [23], [24]. Our study provides new evidence that IS has a negative effect on ACE2/Ang-(1–7)/Mas receptor axis. IS accumulates in renal tubular cells via OAT3-mediated uptake (Fig. 8), and serves as a ligand of AhR in the cytoplasm [20]. The complex of IS-AhR might interact with Stat3, and translocate into the nucleus to recognize and regulate Mas receptor gene other than classical dioxin response elements motifs [25]. However, AhR itself is also a transcription factor, and influences the immune response [26]. It is also possible that AhR alters Mas receptor expression directly without interacting with Stat3. In the present study, we could not discriminate the two pathways. Apart from IS and reactive oxygen species, the increased Ang II in CKD might also act as a negative regulator of ACE2/Ang-(1–7)/Mas receptor axis [27]. Taken together, the effects of IS on RAS and CKD progression are extensive and deleterious.

To confirm the alteration of Mas receptor and its signaling, peNOS was selected to represent the activation of Mas receptor. Our results clearly demonstrated that the change of activated eNOS was completely in parallel with that of Mas receptor. The importance of eNOS in tubular epithelium is rarely discussed. NO produced by eNOS is a paracrine factor to regulate NaCl absorption in the proximal tubules [28], suggesting its involvement in the glomerulotubular feedback and glomerular filtration rate [29]. Reduction of activated eNOS in renal tubules might alter renal microcirculatory dynamic, which then exacerbates renal microenvironmental ischemia [30]. In human study, elevated eNOS expression in the renal vessels and tubules is associated with recovery from ischemia [31]. Furthermore, eNOS inhibits cellular senescence [32], and reduces oxidative stress. Downregulation of Mas receptor/peNOS by IS might be responsible for the renal toxicity of IS, such as oxidative stress, cellular senescence, and abnormal oxygen consumption [14], [33].

In the present study, Ang-(1–7) inhibited IS-induced production of TGF-β1. This finding highlightens the importance of Ang-(1–7)/Mas receptor in the IS-induced renal injury. Administration of Ang-(1–7) has been reported to be beneficial in adriamycin-related kidney failure, 5/6-nephrectomized mice, and experimental diabetes [34]. Ang-(1–7)/Mas receptor axis contributes to their renoprotective effects, at least in part, by counteracting Ang II [35]. Like most of the ACE inhibitors and Ang II type 1 receptor blockers, Ang-(1–7) might provide protective effects independent of blood pressure lowering [36]. The possible mechanism includes modulation of oxidative stress, inflammation, and fibrosis [34]. Ang-(1–7) is also effective in inhibiting IS-related renal toxicity. However, Ang-(1–7) has been shown to exacerbate renal disease with increased ACE activity [8], [37]. This might be due to divergent roles of Ang-(1–7)/Mas receptor in renal cell types such as mesangial cells vs. tubular cells. In addition, treatment dose may be another factor affecting the outcome. Because Ang-(1–7) is metabolized by ACE, overdose of Ang-(1–7) activates ACE [37] and subsequently Ang II pathway. The present study also showed that higher doses of Ang-(1–7) did not provide a better effect in inhibiting TGF-β1 production. The abundance of Mas receptor in the kidney might determine the effect of Ang-(1–7). Thus, reduction of Mas receptor is speculated to accelerate nephron loss in CKD. Novel drugs which stimulate expression and activation of Mas receptor should be developed for the treatment of CKD patients.

In conclusion, Mas receptor expression is reduced in the kidney of CKD rats. IS downregulates renal expression of Mas receptor via OAT3/AhR/Stat3 pathway in proximal tubular cells. IS-induced downregulation of Mas receptor might be involved in upregulation of TGF-β1 in proximal tubular cells.
